# Supplementary material for: Hops across Continents: Exploring How Terroir Transforms the Aromatic Profiles of Five Hop (Humulus lupulus) Varieties Grown in Their Countries of Origin and in Brazil
Source: Plants (Basel). 2024 Sep 24;13(19):2675. doi: 10.3390/plants13192675 (PMC11478771; doi:10.3390/plants13192675)
Supplement: Supplementary file 1 [file plants-13-02675-s001.zip › plants-3210069-supplementary.pdf]

Table S1. Quantification of volatile compounds by Area% identified in the hops (*Humulus lupulus*) variety of Hallertauer Mittelfruher, Magnum, Nugget, Saaz, and Sorachi Ace, planted in Germany (DE), United States (USA), Czech Republic (CZ) and Brazil (BR).

Compound	CAS #	Odor		Flavor	Hallertauer Mittelfruher		Magnum		Nugget		Saaz		Sorachi Ace	
		Type	Strenght	Type	DE (Area%)	BR (Area%)	DE (Area%)	BR (Area%)	USA (Area%)	BR (Area%)	CZ (Area%)	BR (Area%)	USA (Area%)	BR (Area%)
Propan-2-one	67-64-1	Solvent	High		0.006 ± 0.005				0.010 ± 0.001		0.007 ± 0.005		0.010 ± 0.001	
Methyl 2-methylpropanoate	547-63-7	Fruity		Ethereal	0.010 ± 0.001		0.010 ± 0.001		0.010 ± 0.001					
(Methyldisulfanyl)methane	624-92-0	Sulfurous		Sulfurous	0.013 ± 0.005		0.030 ± 0.005		0.030 ± 0.005		0.014 ± 0.005			
2,2,4,6,6-pentamethylheptane	13475-82-6				0.090 ± 0.008									
2,6,6-trimethylbicyclo[3.1.1]hept-2-ene	80-56-8	Herbal	High	Woody	0.083 ± 0.012*	0.020 ± 0.001*	0.140 ± 0.005*	0.130 ± 0.012*	0.200 ± 0.012*	0.050 ± 0.005*		0.074 ± 0.005	0.200 ± 0.008*	0.060 ± 0.008*
2-methylbutan-1-ol	137-32-6	Ethereal	Medium	Ethereal	0.093 ± 0.012		0.110 ± 0.008				0.027 ± 0.005*	0.134 ± 0.005*		
2-methylbut-3-en-2-ol	115-18-4	Herbal				0.093 ± 0.020	0.040 ± 0.005	0.010 ± 0.001	0.011 ± 0.029	0.040 ± 0.005	0.144 ± 0.041	0.137 ± 0.025	0.320 ± 0.078*	0.020 ± 0.008*
Ethyl propanoate	105-37-3	Fruity	High	Fruity				0.001 ± 0.001						
Methyl 2-methylbutanoate	868-57-5	Fruity		Fruity			0.001 ± 0.001		0.010 ± 0.005					
Methyl 3-methylbutanoate	556-24-1	Fruity	Medium	Fruity			0.001 ± 0.005							
2,2-dimethyldecane	17302-37-3										0.214 ± 0.005			
2,2,4,6,6-pentamethylheptane	13475-82-6								0.090 ± 0.001					
(3E)-nona-1,3-diene	56700-77-7								0.010 ± 0.001					0.020 ± 0.001
(2S)-2-methylbutan-1-ol	1565-80-6							0.120 ± 0.005	0.150 ± 0.005*	0.070 ± 0.012*			0.046 ± 0.005*	0.087 ± 0.005*
Camphene	79-92-5	Woody	Medium	Camphoreous					0.110 ± 0.005		0.057 ± 0.005		0.063 ± 0.005	
Butyl nitrite	544-16-1								0.010 ± 0.001					
Pentan-1-ol	71-41-0	Fermented		Fusel					0.001 ± 0.001					
(1S,4R)-2,2-dimethyl-3-methylidenebicyclo[2.2.1]heptane	5794-04-7													0.037 ± 0.005
4-methylpent-3-en-2-one	141-79-7	Vegetable		Potato									0.010 ± 0.001	
6,6-dimethyl-2-methylidenebicyclo[3.1.1]heptane	127-91-3	Herbal	High	Pine	0.603 ± 0.061*	0.170 ± 0.033*	0.890 ± 0.072		0.070 ± 0.009*	0.280 ± 0.046*	0.377 ± 0.005*	30.510 ± 2.668*	0.713 ± 0.012*	0.294 ± 0.017*
2-methylpropyl propanoate	540-42-1	Fruity		Fruity				0.010 ± 0.005	0.120 ± 0.005	0.120 ± 0.022		0.010		0.010 ± 0.001
5-methylhexan-3-one	623-56-3													0.014 ± 0.005
2-methylpropyl 2-methylpropanoate	97-85-8	Fruity		Fruity				0.350 ± 0.065				0.434 ± 0.026	0.020 ± 0.001	0.117 ± 0.005
3-methylbut-2-en-1-ol	556-82-1	Fruity		Fruity								0.010 ± 0.001	0.043 ± 0.017	
7-methyl-3-methylideneocta-1,6-diene	123-35-3	Spicy	High	Woody		20.230 ± 0.849	3.260 ± 0.230	2.520 ± 1.261	0.020 ± 0.009*	0.030 ± 0.005*	27.177 ± 1.170*	0.024 ± 0.005*	12.263 ± 3.963*	24.030 ± 0.070*
3,4-dimethyldecane	17312-45-7					0.030 ± 0.001								
Methyl hexanoate	106-70-7	Fruity	Medium	Fruity			0.020 ± 0.005		0.030 ± 0.005	0.030 ± 0.001			0.010 ± 0.001	
3-methylbut-2-enal	107-86-8	Fruity		Fruity							0.040 ± 0.008			
2-methylpropyl 2-methylpropanoate	97-85-8	Fruity		Fruity	0.140 ± 0.016									
3-methylbut-2-en-1-ol	556-82-1	Fruity		Fruity	0.027 ± 0.005									
[(2E)-3,7-dimethylocta-2,6-dienyl] 3-methylbutanoate	109-20-6	Fruity	Medium	Green	1.877 ± 0.830				2.120 ± 0.416					
2-methyl-5-propan-2-ylcyclohexa-1,3-diene	99-83-2	Terpenic	Medium	Terpenic	0.017 ± 0.005		0.020 ± 0.005*	0.020 ± 0.001*	0.010 ± 0.001			0.017 ± 0.005		0.020 ± 0.001
3,7,7-trimethylbicyclo[4.1.0]hept-2-ene	554-61-0				0.010 ± 0.001						0.010 ± 0.001			
1-methylidene-4-prop-1-en-2-ylcyclohexane	499-97-8				0.010 ± 0.001		0.010 ± 0.008	0.010 ± 0.001				0.007 ± 0.005		0.007 ± 0.005
Methyl hexanoate	106-70-7	Fruity	Medium	Fruity	0.010 ± 0.001	0.037 ± 0.005								
Methyl 4-methylpentanoate	2412-80-8	Fruity		Fruity				0.110 ± 0.008		0.130 ± 0.012				0.020 ± 0.001
Butyl 2-methylpropanoate	97-87-0	Fruity		Fruity								0.027 ± 0.005		
1-methyl-4-propan-2-ylcyclohexa-1,3-diene	99-86-5	Woody	Medium	Terpenic			0.020 ± 0.318*	0.010 ± 0.005*	0.020 ± 0.001			0.014 ± 0.009	0.007 ± 0.005	0.010 ± 0.001
Undec-1-ene	821-95-4												0.010 ± 0.001	0.001 ± 0.001
4-methyl-5-propylnonane	62185-55-1									0.001 ± 0.001				
(4R)-1-methyl-4-prop-1-en-2-ylcyclohexene	5989-27-5	Citrus	Medium	Citrus	0.423 ± 0.037*	0.277 ± 0.009*	0.670 ± 0.016	0.650 ± 0.005	1.070 ± 0.029*	0.420 ± 0.028*	0.744 ± 0.021	0.707 ± 0.046	1,036 ± 0.026*	0.447 ± 0.026*
4,5-dimethylnonane	17302-23-7					0.020 ± 0.001								
S-methyl 3-methylbutanethioate	23747-45-7	Cheesy		Fermented		0.017 ± 0.005	0.020 ± 0.012	0.060 ± 0.005		0.020 ± 0.005		0.017 ± 0.001	0.010 ± 0.001	0.007 ± 0.005
3,5-dimethyl-1,6-heptadiene	68701-99-5										0.010 ± 0.001			
Ethyl 4-methylpentanoate	25415-67-2	Fruity					0.040 ± 0.001							
4-methylidene-1-propan-2-ylbicyclo[3.1.0]hexane	3387-41-5	Woody		Woody				0.530 ± 0.049						
(Z)-9-methylundec-5-ene	74630-65-2										0.010 ± 0.001			
Heptan-2-one	110-43-0	Cheesy	High	Cheesy									0.017 ± 0.005	
3-methylidene-6-propan-2-ylcyclohexene	555-10-2	Minty	Medium		0.057 ± 0.017*	0.107 ± 0.005*	0.210 ± 0.036		0.030 ± 0.012		0.044 ± 0.012	0.217 ± 0.012	0.003± 0.001*	0.260 ± 0.008*
2-methylbutyl propanoate	2438-20-2	Fruity							0.010 ± 0.001					
3-methylbutyl propanoate	105-68-0	Fruity	Medium	Fruity									0.010 ± 0.005	
Pentan-2-yl propanoate	54004-43-2				0.033 ± 0.005*	0.047 ± 0.017*	0.280 ± 0.005*	0.580 ± 0.024*		0.320 ± 0.016		0.624 ± 0.004	0.060 ± 0.001*	0.397 ± 0.012*
Methyl (E)-4-methylpent-2-enoate	50652-78-3				0.001 ± 0.001*	0.013 ± 0.005*			0.001 ± 0.001*	0.010 ± 0.001*	0.001 ± 0.001			
2-methylpropyl 2-methylbutanoate	2445-67-2	Fruity		Fruity								0.114 ± 0.005		0.077 ± 0.026
2,6,6-trimethylbicyclo[3.1.1]hept-2-ene	80-56-8	Herbal	High	Woody	0.093 ± 0.009									
2-methylpropyl 3-methylbutanoate	589-59-3	Fruity	Medium	Green	0.020 ± 0.001									
(3E)-3,7-dimethylocta-1,3,6-triene	3779-61-1	Sweet Herbal	Medium			0.023 ± 0.005	0.090 ± 0.038				0.027 ± 0.005		0.040 ± 0.008	
Hexan-1-ol	111-27-3	Herbal		Green		0.017 ± 0.005					0.017 ± 0.005		0.010 ± 0.001	
(Z)-1-ethoxy-4-methylpent-2-ene	51149-75-8				0.120 ± 0.001*	0.203 ± 0.050*								0.060 ± 0.001

(Z)-hex-3-en-1-ol	928-96-1	Green	High	Green							0.020 ± 0.001			
2-methylpropyl 3-methylbutanoate	589-59-3	Fruity	Medium	Green			0.010 ± 0.001*	0.030 ± 0.005*	0.030 ± 0.001*	0.020 ± 0.001*		0.054 ± 0.005		
1-(3,4-difluorophenyl)-3-[2-(4-hydroxypiperidin-1-yl)-2-oxoethyl]imidazolidin-2-one	2050_1-3						0.340 ± 1.004	0.450 ± 0.025	0.620 ± 0.005*	1.260 ± 0.109*		0.874 ± 0.068		
(3E)-3,7-dimethylocta-1,3,7-triene	502-99-8	Fruity	Medium			1.077 ± 0.039			0.490 ± 0.067					1130 ± 0.016
Ethyl hexanoate	123-66-0	Fruity	High	Fruity		0.010 ± 0.001								
(3Z)-3,7-dimethylocta-1,3,6-triene	3338-55-4	Floral	Medium	Green	0.143 ± 0.020					0.920 ± 0.057	0.124 ± 0.012		0.753 ± 0.142	
2-methylbutyl 2-methylpropanoate	2445-69-4	Fruity			0.733 ± 0.034		2.350 ± 0.062*	1.850 ± 0.069*	1.530 ± 0.020		0.064 ± 0.017*	4.147 ± 0.081*	0.383 ± 0.012	1060 ± 0.008
1-methyl-3-propan-2-ylbenzene	535-77-3				0.050 ± 0.001	0.023 ± 0.005					0.087 ± 0.005		0.147 ± 0.012	
Methyl (Z)-octadec-9-enoate	112-62-9	Mild fatty	Low								0.010 ± 0.001		0.014 ± 0.005	
Methyl 4-methylpentanoate	2412-80-8	Fruity		Fruity	0.027 ± 0.005	0.040 ± 0.008								
Dodecane	112-40-3	Alkane			0.023 ± 0.005	0.010 ± 0.008	0.020 ± 0.005		0.030 ± 0.009		0.097 ± 0.009		0.030 ± 0.001	
Ethyl hexanoate	123-66-0	Fruity	High	Fruity				0.070 ± 0.005						0.107 ± 0.005
1-methyl-2-propan-2-ylbenzene	527-84-4						0.030 ± 0.005*	0.020 ± 0.001*	0.090 ± 0.009	0.020 ± 0.005		0.057 ± 0.009		0.014 ± 0.005
4-methyl-methyl ester octanoic acid	15870-7-2						0.040 ± 0.001					0.147 ± 0.009		
(E)-hex-4-en-1-ol	928-92-7	Green		Green										0.017 ± 0.005
Pentyl propanoate	624-54-4	Fruity		Fruity				0.010 ± 0.001		0.010 ± 0.005		0.024 ± 0.005		0.017 ± 0.005
5-methyl-6-methylene-decane	75029-95-7				0.017 ± 0.005		0.010 ± 0.001							
1-methyl-4-propan-2-ylidenecyclohexene	586-62-9	Herbal	Medium	Woody	0.017 ± 0.005		0.030 ± 0.001	0.030 ± 0.005	0.050 ± 0.005*	0.020 ± 0.005*			0.027 ± 0.005	0.027 ± 0.005
Hexyl acetate	142-92-7	Fruity	Medium	Fruity	0.001 ± 0.001			0.010 ± 0.001		0.010 ± 0.001		0.034 ± 0.014		0.010 ± 0.001
Di(imidazol-1-yl)methanone	530-62-1													0.017 ± 0.005
4-pentenyl ester butanoic acid	30563-31-6							0.010 ± 0.001						
Pentyl 2-methylpropanoate	2445-72-9	Fruity			0.010 ± 0.001		0.010 ± 0.001	0.010 ± 0.001	0.010 ± 0.001	0.020 ± 0.001		0.047 ± 0.009	0.003 ± 0.001	0.020 ± 0.001
Dodec-1-ene	112-41-4												0.010 ± 0.001	
Methyl heptanoate	106-73-0	Fruity		Fruity	0.047 ± 0.005*	0.670 ± 0.051*	0.040 ± 0.001*	0.050 ± 0.005*	0.060 ± 0.005*	0.160 ± 0.012*		0.104 ± 0.012		0.007 ± 0.005
Methyl 2-methylheptanoate	51209-78-0				0.020 ± 0.001	0.043 ± 0.005								
Ethyl (E)-hex-3-enoate	2396-83-0	Fruity	Medium	Fruity										0.027 ± 0.005
2-methylbutyl butanoate	51115-64-1	Fruity		Fruity										0.020 ± 0.001
methyl 2-methylheptanoate	51209-78-0						0.010 ± 0.005			0.020 ± 0.001	0.040 ± 0.001	0.034 ± 0.005		
6-methylhept-2-en-4-one	49852-35-9						0.001 ± 0.001							
5-methylheptan-2-ol	54630-50-1							0.001 ± 0.001						
Octan-2-one	111-13-7	Earthy	Medium	Dairy	0.001 ± 0.001*	0.027 ± 0.005*	0.001 ± 0.001			0.010 ± 0.001	0.030 ± 0.001		0.010 ± 0.001	
Methyl (E)-5-methylhex-2-enoate	68797-67-1								0.020 ± 0.001		0.010 ± 0.001			
[(Z)-hex-3-enyl] acetate	3681-71-8	Green	High	Green								0.007 ± 0.005		
Ethyl 5-methylhexanoate	10236-10-9							0.070 ± 0.005						
3-methylbut-2-enyl 2-methylpropanoate	76649-23-5	Fruity			0.030 ± 0.001	0.023 ± 0.005	0.010 ± 0.001*	0.060 ± 0.005*		0.030 ± 0.001			0.010 ± 0.001*	0.030 ± 0.001*
4-methylene-methyl ester hexanoic acid	73805-48-8				0.733 ± 0.019	0.803 ± 0.059	0.470 ± 0.005		0.620 ± 0.017*	0.740 ± 0.035*	0.887 ± 0.017*	0.517 ± 0.024*	0.034 ± 0.005*	0.060 ± 0.001*
Oct-1-en-3-ol	3391-86-4	Earthy	High	Mushroom		0.050 ± 0.008					0.087 ± 0.005*	0.104 ± 0.005*	0.024 ± 0.005*	0.054 ± 0.005*
6-methylhept-5-en-2-one	110-93-0	Citrus	Medium	Green		0.143 ± 0.024					0.020 ± 0.022		0.217 ± 0.020	
Heptan-1-ol	111-70-6	Green	Medium	Solvent										0.027 ± 0.005
Methyl (E)-hept-2-enoate	22104-69-4							0.460 ± 0.019						
3-methylbutanoic acid	503-74-2	Cheesy	High	Cheesy	0.460 ± 0.064		0.110 ± 0.005		0.220 ± 0.014		0.127 ± 0.019		0.567 ± 0.041	
3-methylbutyl 2-methylbutanoate	27625-35-0	Fruity		Fruity			0.030 ± 0.005	0.030 ± 0.001	0.060 ± 0.001	0.040 ± 0.005				
2-methylbutyl 2-methylbutanoate	2445-78-5	Fruity		Fruity			0.250 ± 0.008	0.190 ± 0.016	0.300 ± 0.012	0.150 ± 0.017		0.457 ± 0.045		0.160 ± 0.001
2-methylbutyl 3-methylbutanoate	2445-77-4	Fruity		Fruity		0.053 ± 0.009	0.150 ± 0.005	0.180 ± 0.009	0.360 ± 0.014	0.190 ± 0.017				
3-methylpentanoic acid	105-43-1	Animal	Medium	Sour				0.060 ± 0.005						0.044 ± 0.005
3-methylbutyl 3-methylbutanoate	659-70-1	Fruity	Medium	Green						0.090 ± 0.019		0.137 ± 0.052		
2-methylbutanoic acid	116-53-0	Acidic	Medium	Fruity							0.057 ± 0.012			
Methyl 6-methylheptanoate	2519-37-1				0.070 ± 0.001*	0.920 ± 0.071*	0.140 ± 0.001	0.560 ± 0.029	0.120 ± 0.001	0.770 ± 0.048	0.020 ± 0.001	1.217 ± 0.074	0.020 ± 0.001*	0.070 ± 0.001*
4-methyl-1-prop-1-en-2-ylcyclohexene	586-67-4				0.033 ± 0.005									
[(Z)-hex-3-enyl] butanoate	16491-36-4	Green	Medium	Green				0.010 ± 0.001		0.010 ± 0.005		0.080 ± 0.014		
Methyl (2S,4R)-2,4-dimethylheptanoate	18450-78-7				0.003 ± 0.005									
Methyl 7-methyloctanoate	2177-86-8					0.017 ± 0.005	0.010 ± 0.001			0.010 ± 0.001	0.130 ± 0.022			
Methyl (2S,4S)-2,4-dimethylhexanoate	14251-45-7							0.010 ± 0.005						
Methyl 2,4-dimethylnonanoate	54889-61-1										0.010 ± 0.001			
Ethyl heptanoate	106-30-9	Fruity	Medium	Fruity				0.070 ± 0.005						0.020 ± 0.001
Methyl (2S,4R)-2,4-dimethylheptanoate	18450-78-7											0.020 ± 0.001		
(E)-1-cyclohexylbut-2-en-1-ol	79605-62-2												0.034 ± 0.005	
3-(4-methylpent-3-enyl)furan	539-52-6	Woody	Medium		0.170 ± 0.022*	0.027 ± 0.009*	0.120 ± 0.005*	0.020 ± 0.005*	0.210 ± 0.016*	0.040 ± 0.012*	0.230 ± 0.014*	0.097 ± 0.047*	0.430 ± 0.037*	0.024 ± 0.005*
Hexyl propanoate	2445-76-3	Fruity						0.020 ± 0.005		0.040 ± 0.001				0.070 ± 0.001
Hexahydro-1,1-dimethyl-4-methylene-4H-cyclopenta[c]furan	344294-72-0				0.133 ± 0.005		0.050 ± 0.005*	0.010 ± 0.001*	0.100 ± 0.005*	0.030 ± 0.005*	0.154 ± 0.005*	0.060 ± 0.008*	0.237 ± 0.001*	0.020 ± 0.001*
1,5,5-trimethyl-6-methylidenecyclohexene	514-95-4						0.040 ± 0.001							
2-methyl-6-methylene-2-octene	10054-9-8										0.027 ± 0.009			
3,3-dimethylcyclohexan-1-one	2979-19-3										0.070 ± 0.008			

5,9-dimethyl-, (E)-5,8-decadien-2-one	130876-99-2				0.047 ± 0.033								0.127 ± 0.005	
(4E,6Z)-2,6-dimethylocta-2,4,6-triene	7216-56-0							0.010 ± 0.001						0.024 ± 0.005
S-ethyl hexanethioate	2450_12-6					0.050 ± 0.008								
2-[(2R,5R)-5-ethenyl-5-methyloxolan-2-yl]propan-2-ol	34995-77-2	Floral	Medium		0.107 ± 0.005	0.060 ± 0.008					0.040 ± 0.008		0.087 ± 0.012	
Heptyl ester acetic acid	112-6-1						0.001 ± 0.005	0.010 ± 0.001	0.010 ± 0.001	0.010 ± 0.001		0.037 ± 0.005		0.010 ± 0.001
S-propyl hexanethioate	2432-78-2									0.001 ± 0.001				0.027 ± 0.005
[(Z)-hex-3-enyl] 2-methylbutanoate	53398-85-9	Green	Medium	Green								0.007 ± 0.005		
hexyl 2-methylpropanoate	2349_7-7						0.040 ± 0.001*	0.020 ± 0.001*	0.070 ± 0.008*	0.120 ± 0.008*		0.130 ± 0.008		0.090 ± 0.001
Methyl octanoate	111-11-5	Waxy		Green	0.050 ± 0.001*	0.777 ± 0.054*	0.070 ± 0.001*	0.090 ± 0.008*	0.150 ± 0.005*	0.500 ± 0.034*		0.284 ± 0.019		0.024 ± 0.005
3-methoxybutan-2-ol	53778-72-6										0.030 ± 0.001		0.044 ± 0.005	
2-methylpropyl hexanoate	105-79-3	Fruity	Medium	Fruity	0.010 ± 0.001*	0.017 ± 0.005*							0.001 ± 0.001	
Benzaldehyde	100-52-7	Fruity	High	Fruity	0.017 ± 0.005*	0.013 ± 0.005*			0.020 ± 0.001		0.020 ± 0.001*	0.044 ± 0.005*	0.054 ± 0.005*	0.027 ± 0.005*
2,6-dimethyl-1,3,5,7-octatetraene, E,E-	460-1-5									0.010 ± 0.001				
[(Z)-hex-2-enyl] acetate	56922-75-9							0.020 ± 0.001						
Ethyl octanoate	106-32-1	Waxy	Medium	Waxy				0.110 ± 0.005			0.010 ± 0.001			0.020 ± 0.001
Methyl 7-methyloctanoate	2177-86-8						0.010 ± 0.001							
2,2-dimethyl-3-[(2E)-3-methylpenta-2,4-dienyl]oxirane	28977-57-3				0.030 ± 0.008		0.030 ± 0.009		0.040 ± 0.012		0.070 ± 0.022		0.027 ± 0.012	
[(E)-hex-3-enyl] 2-methylpropanoate	84682-20-2					0.010 ± 0.001						0.030 ± 0.008		
Cyclohept-4-en-1-ol	38607-27-1					0.037 ± 0.005				0.020 ± 0.009		0.087 ± 0.052		
2-methylpent-4-en-1-ol	5673-98-3											0.134 ± 0.005		
Nonan-2-one	821-55-6	Fruity	Medium	Cheesy	0.447 ± 0.009*	0.813 ± 0.060*	0.170 ± 0.005*	0.070 ± 0.001*	0.090 ± 0.005*	0.026 ± 0.009*	0.827 ± 0.033		0.554 ± 0.037*	0.627 ± 0.017*
4-hydroxyhexan-3-one	4984-85-4				0.070 ± 0.001		0.030 ± 0.005		0.030 ± 0.005		0.017 ± 0.005*	0.040 ± 0.001*	0.027 ± 0.005*	0.017 ± 0.005*
Methyl ester 4-octenoic acid	1732-0-9					0.033 ± 0.005								
Nonan-2-ol	628-99-9	Waxy		Waxy	0.080 ± 0.001				0.040 ± 0.005	0.040 ± 0.001	0.124 ± 0.005		0.020 ± 0.001	0.010 ± 0.001
Trans-propionate 2-methyl-cyclohexanol	15287-79-3							0.020 ± 0.005			0.027 ± 0.001			
Octan-1-ol	111-87-5	Waxy	Medium	Waxy	0.047 ± 0.005*	0.107 ± 0.012*	0.040 ± 0.005*	0.030 ± 0.001*	0.030 ± 0.005*	0.060 ± 0.005*	0.020 ± 0.001*	0.060 ± 0.001*	0.017 ± 0.005*	0.064 ± 0.005*
5-pentylcyclohexa-1,3-diene	56318-84-4						0.001 ± 0.001							
3,7-dimethylocta-1,6-dien-3-ol	78-70-6	Floral	Medium	Citrus	2.86 ± 0.043*	2.033 ± 0.105*	0.810 ± 0.017*	0.620 ± 0.029*	2.070 ± 0.071*	1.650 ± 0.050*	1.804 ± 0.063*	1.590 ± 0.036*	0.964 ± 0.034*	0.764 ± 0.034*
[(1R,2R)-2-methylcyclohexyl] butanoate	15287-80-6							0.010 ± 0.001		0.010 ± 0.001				
Methyl 7-methyloctanoate	5129-53-3					0.053 ± 0.005	0.050 ± 0.001	0.110 ± 0.008		0.050 ± 0.001		0.234 ± 0.012	0.010 ± 0.001	0.010 ± 0.001
3,5,5-trimethyl-2H-furan	23230-79-7					0.070 ± 0.008								
[1-(hydroxymethyl)cyclohexyl]methanol	2658-60-8												0.017 ± 0.005	
Ethyl octanoate	106-32-1	Waxy	Medium	Waxy				0.110 ± 0.008					0.017 ± 0.005	0.020 ± 0.001
6-methyl-7-octen-2-one	35215-49-7					0.017 ± 0.005								
Methyl 6-methyloctanoate	5129-62-4				0.010 ± 0.001*	0.067 ± 0.012*	0.050 ± 0.001*	0.270 ± 0.009*	0.010 ± 0.005*	0.200 ± 0.012*			0.010 ± 0.001*	0.024 ± 0.005*
Heptyl propanoate	2216-81-1	Floral		Fruity			0.010 ± 0.001*	0.040 ± 0.005*		0.030 ± 0.005			0.020 ± 0.001*	0.064 ± 0.001*
(1R,4R)-1-methyl-4-propan-2-ylcyclohex-2-en-1-ol	29803-82-5					0.013 ± 0.005				0.010 ± 0.005				0.010 ± 0.001
(6Z)-7,11-dimethyl-3-methylidenedodeca-1,6,10-triene	28973-97-9	Green			0.020 ± 0.001									
(E)-oct-2-en-4-ol	4798-61-2				0.027 ± 0.005									
(4S)-1-methyl-4-(6-methylhepta-1,5-dien-2-yl)cyclohexene	495-61-4	Balsamic						0.020 ± 0.001						
Octyl acetate	112-14-1	Floral	Medium	Waxy	0.007 ± 0.005	0.010 ± 0.001	0.010 ± 0.001			0.010 ± 0.005		0.074 ± 0.005		0.010 ± 0.001
1,3,3-trimethylbicyclo[2.2.1]heptan-2-ol	1632-73-1	Camphoreous	Medium	Camphoreous							0.034 ± 0.005			
Heptyl 2-methylpropanoate	2349-13-5	Fruity		Berry	0.173 ± 0.009		0.120 ± 0.005		0.040 ± 0.001	0.150 ± 0.009		0.120 ± 0.001	0.044 ± 0.005*	0.207 ± 0.009*
Nonan-2-yl acetate	14936-66-4							0.030 ± 0.001						
(1R,5S,6R,7S,10R)-4,10-dimethyl-7-propan-2-yltricyclo[4.4.0.01,5]dec-3-ene	17699-14-8	Herbal				0.037 ± 0.005		0.200 ± 0.008						
S-methyl heptanethioate	2432-82-8					0.033 ± 0.005								
Methyl nonanoate	1731-84-6	Fruity		Winey	0.040 ± 0.008*	0.677 ± 0.041*	0.060 ± 0.001*	0.070 ± 0.005*	0.080 ± 0.001*	0.310 ± 0.009*	0.024 ± 0.005*	0.300 ± 0.022*		0.020 ± 0.008
(E)-2-(6,10-dimethylundeca-1,5,9-trien-2-yl)oxirane	83637-40-5				0.177 ± 0.029	0.137 ± 0.069	0.330 ± 0.017	0.310 ± 0.322	0.090 ± 0.001	0.230 ± 0.336	0.177 ± 0.009	0.647 ± 0.603	0.137 ± 0.012	0.220 ± 0.123
Hexanoic acid	142-62-1	Fatty	Medium	Cheesy	0.060 ± 0.008*	0.027 ± 0.009*	0.040 ± 0.008		0.080 ± 0.009		0.024 ± 0.005		0.110 ± 0.008*	0.037 ± 0.009*
2-methylbutyl hexanoate	2601-13-0	Ethereal			0.027 ± 0.005*	0.043 ± 0.005*	0.020 ± 0.001	0.020 ± 0.005	0.040 ± 0.001	0.020 ± 0.001				0.014 ± 0.005
2-methyl-6-methylideneocta-1,7-dien-3-one	41702-60-7													0.067 ± 0.012
Methyl non-3-enoate	13481-87-3	Fruity	Medium	Fruity		0.327 ± 0.019				0.270 ± 0.008				
Methyl non-4-enoate	20731-19-5							0.120 ± 0.054				0.760 ± 0.149		
5-Nonenoic acid methyl ester	20731-20-8								0.090 ± 0.009					
(1S,2R,6R,7R,8S)-1,3-dimethyl-8-propan-2-yltricyclo[4.4.0.02,7]dec-3-ene	14912-44-8				0.320 ± 0.014*	0.077 ± 0.005*	0.240 ± 0.014*	0.330 ± 0.059*	0.420 ± 0.017*	0.120 ± 0.005*	0.246 ± 0.021*	0.124 ± 0.005*	0.384 ± 0.019*	0.230 ± 0.008*
(1R,2S,6S,7S,8S)-1,3-dimethyl-8-propan-2-yltricyclo[4.4.0.02,7]dec-3-ene	3856-25-5	Woody			0.933 ± 0.025*	0.217 ± 0.020*	0.730 ± 0.025*	1.270 ± 0.033*	1.680 ± 0.050*	0.310 ± 0.022*	0.700 ± 0.037*	0.300 ± 0.022*	1.424 ± 0.070*	0.814 ± 0.017*
Decan-2-one	693-54-9	Floral	Medium	Fermented	0.740 ± 0.049*	0.833 ± 0.046*	0.280 ± 0.012*	0.030 ± 0.005*	0.010 ± 0.001*	0.410 ± 0.020*	1.120 ± 0.062*	0.127 ± 0.009*	0.880 ± 0.045*	0.447 ± 0.031*
2-Methyl-2-(4-methyl-3-pentenyl)-cyclopropanemethanol	98678-70-7												0.067 ± 0.005	
Decyl trifluoroacetate	333-88-0								0.060 ± 0.005*	0.030 ± 0.001*	0.067 ± 0.005			
7-Decen-2-one	35194-33-3				0.067 ± 0.005*	0.110 ± 0.008*	0.020 ± 0.001				0.187 ± 0.021		0.044 ± 0.009	0.040 ± 0.014
3,7-dimethylocta-2,6-dienyl acetate	16409-44-2				0.020 ± 0.001								0.024 ± 0.012	
5,5-dimethylfuran-2-one	20019-64-1				0.203 ± 0.005		0.040 ± 0.005						0.170 ± 0.008	
7-epi-sesquithujene	159407-35-9					0.037 ± 0.005								

2-methyl-6-methylideneocta-2,7-dien-4-ol	14434-41-4							0.030 ± 0.005				0.080 ± 0.016		
4,6-dimethyloctanoic acid	2553-96-0								0.040 ± 0.001			0.074 ± 0.031		
7-methyl-3-methylideneoct-6-enal	55050-40-3	Aldehydic	Medium		0.183 ± 0.005*	0.083 ± 0.005*	0.060 ± 0.008			0.090 ± 0.012	0.254 ± 0.033*	0.170 ± 0.014*	0.274 ± 0.012	
3,5-Dimethyl-1,6-heptadiene	68701-99-5													0.054 ± 0.012
(E)-2-methylpent-2-enoic acid	16957-70-3	Fruity	Medium										0.030 ± 0.001	
(1S,2R,4S)-1,7,7-trimethylbicyclo[2.2.1]heptan-2-ol	464-45-9	Balsamic	Medium	Camphoreous					0.030 ± 0.005		0.037 ± 0.009			
(Methyldisulfanyl)-methylsulfanylmethane	42474-44-2	Sulfurous						0.060 ± 0.008						
5-methylhexanoic acid	628-46-6	Fatty	Medium				0.060 ± 0.005*	0.050 ± 0.008*						
Octyl propanoate	142-60-9	Fruity		Estery			0.020 ± 0.001			0.020 ± 0.001		0.037 ± 0.009		0.024 ± 0.005
(1S,2S,6R,8S)-2,8-dimethyl-6-prop-1-en-2-yltricyclo[4.4.0.02,8]decane	87064-18-4							0.080 ± 0.009						
1,7,7-trimethylbicyclo[2.2.1]heptan-2-ol	507-70-0	Balsamic											0.027 ± 0.009	0.020 ± 0.008
(5E)-2,6-dimethylocta-1,5,7-trien-3-ol	29414-56-0	Camphoreous	Medium		0.157 ± 0.029	0.210 ± 0.061	0.300 ± 0.017	0.350 ± 0.312	0.060 ± 0.009	0.270 ± 0.375	0.204 ± 0.031	0.737 ± 0.695	0.107 ± 0.012	0.214 ± 0.120
Octyl 2-methylpropanoate	109-15-9	Waxy	Medium	Creamy		0.023 ± 0.005	0.060 ± 0.001							0.040 ± 0.014
(2S)-5-[[amino(phenylmethoxycarbonylamino)methylidene]amino]-2-(phenylmethoxycarbonylamino)pentanoic acid	53934-75-1													0.410 ± 0.049
Pentadec-1-ene	13360-61-7						0.120 ± 0.019		0.130 ± 0.034					
Heptanoic acid	111-14-8	Cheesy		Waxy					0.070 ± 0.034					
Prop-2-enyl 3-methylbutanoate	2835-39-4	Fruity		Fruity		0.017 ± 0.009								
(1S,4aR,7R)-1,4a-Dimethyl-7-(prop-1-en-2-yl)-1,2,3,4,4a,5,6,7-octahydronaphthalene	52026-55-8					0.143 ± 0.019								
Methyl (2Z)-3,7-dimethylocta-2,6-dienoate	1862-61-9	Floral			0.043 ± 0.005		0.020 ± 0.005				0.027 ± 0.005			
(1S,2S,4R)-1-ethenyl-1-methyl-2,4-bis(prop-1-en-2-yl)cyclohexane	515-13-9	Sweet	Medium			0.043 ± 0.005								
(1S,5S,6R)-2,6-dimethyl-6-(4-methylpent-3-enyl)bicyclo[3.1.1]hept-2-ene	13474-59-4	Woody				2.673 ± 0.049					2.084 ± 0.170			
Bicyclo[3.1.1]hept-2-ene, 2,6-dimethyl-6-(4-methyl-3-pentenyl)-	17699-5-7								0.310 ± 0.012			0.904 ± 0.026		
Methyl decanoate	110-42-9	Fermented		Fatty						0.660 ± 0.160				
2-(2,4-difluorophenyl)-1-[4-[6-(4-methylpiperazin-1-yl)pyridazin-3-yl]piperazin-1-yl]ethanone	1191-2-2				0.680 ± 0.059*	2.243 ± 0.108*	0.710 ± 0.017*	1.420 ± 0.018*	0.570 ± 0.025*	3.670 ± 0.257*	0.127 ± 0.005*	2.527 ± 0.111*		0.134 ± 0.032
(1R,2S)-1-methyl-3-methylidene-8-propan-2-yltricyclo[4.4.0.02,7]decane	18252-44-3					0.067 ± 0.024	0.060 ± 0.005	0.090 ± 0.017			0.134 ± 0.026	0.117 ± 0.009		
Undecan-2-one	112-12-9	Fruity	Medium	Waxy	12.033 ± 0.229*	0.503 ± 0.020*	8.460 ± 0.249*	7.190 ± 0.187*	11.930 ± 0.316*	8.460 ± 0.283*	8.814 ± 0.462*	4.350 ± 0.135*	16.464 ± 0.571*	12.120 ± 0.156*
Tetradec-1-ene	1120-36-1							0.050 ± 0.014						
(1R,4E,9S)-4,11,11-trimethyl-8-methylidenebicyclo[7.2.0]undec-4-ene	87-44-5	Spicy	Medium	Spicy	3.380 ± 0.154		4.340 ± 0.192	4.360 ± 0.109	4.86 ± 0.950	3.34 ± 0.145	2.147 ± 0.127*	2.697 ± 0.087*	3.897 ± 0.471*	4.077 ± 0.182*
(Z)-Undec-6-en-2-one	107853-70-3				0.243 ± 0.012*	0.453 ± 0.020*				0.180 ± 0.034	0.684 ± 0.041*	0.140 ± 0.008*		0.254 ± 0.005
(4aS,9aR)-3,5,5-trimethyl-9-methylidene-2,4a,6,7,8,9a-hexahydro-1H-benzo[7]annulene	3853-83-6				0.027 ± 0.005								0.024 ± 0.005	0.040 ± 0.008
Methyl (11E,14E)-icosa-11,14-dienoate	2463_02-7										0.070 ± 0.001			
Trans-geranic acid methyl ester	1189-9-9				0.500 ± 0.024*	0.397 ± 0.017*	0.570 ± 0.020*	0.290 ± 0.014*	0.360 ± 0.019*	0.310 ± 0.012*	0.250 ± 0.016*	0.304 ± 0.005*	0.180 ± 0.001*	0.087 ± 0.026*
(6Z)-7,11-dimethyl-3-methylidenedodeca-1,6,10-triene	28973-97-9	Green				25.103 ± 1.297		0.060 ± 0.009						
(6E)-7,11-dimethyl-3-methylidenedodeca-1,6,10-triene	18794-84-8	Woody			0.793 ± 0.026					8.080 ± 0.306	13.230 ± 0.334*	6.727 ± 1.073*	0.867 ± 0.124*	4.634 ± 0.022*
(1R,2R)-1-ethenyl-1-methyl-4-propan-2-ylidene-2-prop-1-en-2-ylcyclohexane	29873-99-2	Green	Medium		0.017 ± 0.005				0.040 ± 0.001					
(1S,4S,4aS)-1-Isopropyl-4,7-dimethyl-1,2,3,4,4a,5-hexahydronaphthalene	267665-20-3												8.174 ± 3.111	
6-methylheptanoic acid	929-10-2								0.020 ± 0.001					
4a,8-dimethyl-2-prop-1-en-2-yl-2,3,4,5,6,7-hexahydro-1H-naphthalene	103827-22-1											0.267 ± 0.019		
(2E)-3,7-dimethylocta-2,6-dien-1-ol	106-24-1	Floral	Medium	Floral								0.567 ± 0.087		
Ethyl (E)-dec-4-enoate	76649-16-6	Green	Medium	Fatty				0.240 ± 0.009						
(1E,4E,8E)-2,6,6,9-tetramethylcycloundeca-1,4,8-triene	6753-98-6	Woody			3.930 ± 0.889	3.807 ± 0.351	3.680 ± 1.075	2.800 ± 0.147	5.500 ± 0.190*	4.490 ± 0.287*	8.284 ± 0.641*	9.304 ± 0.738*	3.227 ± 3.150	6.030 ± 0.279
(3Z,6E)-3,7,11-trimethyldodeca-1,3,6,10-tetraene	26560-14-5				0.077 ± 0.005								0.114 ± 0.005*	0.417 ± 0.009*
1,1,7,7a-tetramethyl-2,3,5,6,7,7b-hexahydro-1aH-cyclopropa[a]naphthalene	17334-55-3												0.267 ± 0.005	
(1R,8aS)-1,6-dimethyl-4-propan-2-yl-1,2,3,7,8,8a-hexahydronaphthalene	41702-63-0								0.210 ± 0.016					0.160 ± 0.022
(4S)-1-methyl-4-(6-methylhepta-1,5-dien-2-yl)cyclohexene	495-61-4	Balsamic									0.410 ± 0.028			
Methyl undec-10-enoate	111-81-9	Fatty		Waxy								0.287 ± 0.009		
(1S,4S,7R)-1,4-dimethyl-7-prop-1-en-2-yl-1,2,3,4,5,6,7,8-octahydroazulene	3691_12-1	Woody								0.050 ± 0.008				
(1S,4aS,8aR)-4,7-dimethyl-1-propan-2-yl-1,2,4a,5,6,8a-hexahydronaphthalene	10208-80-7	Woody			0.580 ± 0.049		0.390 ± 0.025		0.550 ± 0.026		0.250 ± 0.014		0.747 ± 0.033*	0.514 ± 0.012*
(1R,4aR,8aS)-7-methyl-4-methylidene-1-propan-2-yl-2,3,4a,5,6,8a-hexahydro-1H-naphthalene	30021-74-0	Woody					0.540 ± 0.025						1.240 ± 0.102	
(3R,4aR,5S)-4a,5-dimethyl-3-prop-1-en-2-yl-2,3,4,5,6,7-hexahydro-1H-naphthalene	10219-75-7							0.080 ± 0.009			0.720 ± 0.043			
[(2E)-3,7-dimethylocta-2,6-dienyl] acetate	105-87-3	Floral	Medium	Green				2.350 ± 0.119		3.510 ± 0.230		3.694 ± 0.120		
Gamma-maalinene	20071-49-2							1.130 ± 0.063						
(3R,4aR,8aR)-5,8a-dimethyl-3-prop-1-en-2-yl-2,3,4,4a,7,8-hexahydro-1H-naphthalene	473-13-2	Amber			0.557 ± 0.005	4.650 ± 0.607			1.590 ± 0.229*	7.810 ± 0.310*		1.240 ± 0.070	0.440 ± 0.014*	0.694 ± 0.034*
4a,8-Dimethyl-2-(prop-1-en-2-yl)-1,2,3,4,4a,5,6,7-octahydronaphthalene	473-14-3				0.020 ± 0.001			0.020 ± 0.012	0.040 ± 0.005					
Naphthalene, 1,2,3,4,4a,5,6,7-octahydro-4a,8-dimethyl-2-(1-methylethenyl)-	103827-22-1					1.157 ± 0.118								
(3R,4aS,8aR)-8a-methyl-5-methylidene-3-prop-1-en-2-yl-1,2,3,4,4a,6,7,8-octahydronaphthalene	17066-67-0	Herbal				10.713 ± 0.572			1.830 ± 0.097*	0.200 ± 0.005*			0.014 ± 0.005	0.020 ± 0.005
,1,4,7-tetramethyl-1a,2,3,4,4a,5,6,7b-octahydrocyclopropa[e]azulene	489-40-7	Woody								0.070 ± 0.009		0.020 ± 0.001		
(3E,6E)-3,7,11-trimethyldodeca-1,3,6,10-tetraene	502-61-4	Woody		Green	0.073 ± 0.005*	0.447 ± 0.070*			0.070 ± 0.005		0.050 ± 0.001		0.084 ± 0.012*	0.264 ± 0.009*
1,1,4,7-tetramethyl-1a,2,3,4,4a,5,6,7b-octahydrocyclopropa[e]azulene	489-40-7	Woody			0.047 ± 0.005*	0.143 ± 0.012*								
(1R,4aS,8aS)-7-methyl-4-methylidene-1-propan-2-yl-2,3,4a,5,6,8a-hexahydro-1H-naphthalene	39029-41-9	Woody	Medium		2.133 ± 0.103		1.250 ± 0.008*	2.210 ± 0.163*	26.35 ± 1.066*	0.690 ± 0.073*	18.644 ± 0.073			1.320
(1S,8aR)-4,7-dimethyl-1-propan-2-yl-1,2,3,5,6,8a-hexahydronaphthalene	483-76-1	Herbal			0.717 ± 0.069	0.690 ± 0.120	0.560 ± 0.049*	0.900 ± 0.198*	0.720 ± 0.080		0.624 ± 0.053*	0.684 ± 0.054*	3.737 ± 0.134	0.624 ± 0.041
(1aR,7R,7aR,7bS)-1,1,7,7a-tetramethyl-2,4,5,6,7,7b-hexahydro-1aH-cyclopropa[a]naphthalene	6831-16-9				0.010 ± 0.001	0.040 ± 0.008		0.040 ± 0.005		0.020 ± 0.005				0.010 ± 0.001

7-epi-alpha-selinene	123123-37-5					0.210 ± 0.016								
(3R)-3-[(2S)-6-methylhept-5-en-2-yl]-6-methylidenecyclohexene	20307-83-9	Herbal	Medium			0.023 ± 0.009								0.010 ± 0.001
(1S,4aR,8aS)-4,7-dimethyl-1-propan-2-yl-1,2,4a,5,8,8a-hexahydronaphthalene	523-47-7	Woody	Medium		0.150 ± 0.008			0.190 ± 0.016						
(4aR,8aR)-5,8a-dimethyl-3-propan-2-ylidene-1,2,4,4a,7,8-hexahydronaphthalene	6813-21-4				0.723 ± 0.052									
1-methyl-4-[(2E)-6-methylhepta-2,5-dien-2-yl]cyclohexene	25532-79-0										0.010 ± 0.001			
Zonarene	41929-5-9												0.163 ± 0.005*	0.134 ± 0.005*
.alpha.-Maaliene	489-28-1									0.180 ± 0.008		0.067 ± 0.005		
1,6-dimethyl-4-propan-2-yl-1,2,3,4,4a,7-hexahydronaphthalene	16728-99-7						0.130 ± 0.005*	1.190 ± 0.106*	0.170 ± 0.012		0.054 ± 0.005		0.143 ± 0.005*	0.287 ± 0.036*
1,2,4a,5,6,8a-hexahydro-4,7-dimethyl-1-(1-methylethyl)-, [1S-(1.alpha.,4a.beta.,8a.alpha.)]-naphthalene	24406-5-1						0.120 ± 0.005		0.150 ± 0.009		0.080 ± 0.008		0.267 ± 0.012	0.127 ± 0.005
3-(1,1-dimethylethyl)-2,5-furandione	18261-7-9				0.013 ± 0.009	0.023 ± 0.005					0.027 ± 0.005		0.037 ± 0.019	0.024 ± 0.005
[(2Z)-3,7-dimethylocta-2,6-dienyl] 2-methylpropanoate	2345-24-6	Fruity	Medium	Fruity					0.020 ± 0.001					
(4-prop-1-en-2-ylcyclohexen-1-yl)methanol	536-59-4	Green	Medium	Woody					0.030 ± 0.005					
[(2Z)-3,7-dimethylocta-2,6-dienyl] 2-methylbutanoate	51117-19-2						0.010 ± 0.001					0.020 ± 0.001		
[(2Z)-3,7-dimethylocta-2,6-dienyl] butanoate	999-40-6	Green		Green	0.020 ± 0.001					0.010 ± 0.001		0.010 ± 0.001	0.013 ± 0.005	0.010 ± 0.001
Methyl (Z)-octadec-6-enoate	2777-58-4									0.040 ± 0.005		0.030 ± 0.001		0.003 ± 0.005
[(4S)-4-prop-1-en-2-ylcyclohexen-1-yl]methanol	18457-55-1				0.047 ± 0.005*	0.010 ± 0.001*	0.020 ± 0.005*	0.010 ± 0.001*		0.020 ± 0.017	0.044 ± 0.009		0.090 ± 0.008	0.010 ± 0.001
2-tridecanone	593-8-8				0.190 ± 0.022*	0.243 ± 0.024*	0.090 ± 0.005*	0.050 ± 0.005*	0.030 ± 0.005*	0.220 ± 0.008*	0.248 ± 0.029*	0.160 ± 0.001*	0.013 ± 0.005*	0.127 ± 0.001*
[(2E)-3,7-dimethylocta-2,6-dienyl] propanoate	105-90-8	Floral	Medium	Waxy		0.060 ± 0.008	0.050 ± 0.001	0.280 ± 0.012		0.060 ± 0.001		0.260 ± 0.016		0.024 ± 0.005
Methyl dodecanoate	111-82-0	Waxy	Medium	Waxy						0.001 ± 0.005				
Trifluoroacetyl-lavandulol	28673-24-7				0.020 ± 0.001									
(1S,4S)-1,6-dimethyl-4-propan-2-yl-1,2,3,4-tetrahydronaphthalene	483-77-2	Herbal Spicy	Medium		0.290 ± 0.024				0.190 ± 0.019		0.134 ± 0.017		0.427 ± 0.037*	0.090 ± 0.021*
[(2E)-3,7-dimethylocta-2,6-dienyl] butanoate	106-29-6	Fruity	Medium	Fruity		0.100 ± 0.016	0.620 ± 0.017*	0.460 ± 0.028*		0.210 ± 0.008				
Benzyl pentanoate	10361-39-4	Fruity											0.004 ± 0.005	0.001 ± 0.001
(Z,Z)-1,8,11-heptadecatriene	56134-3-3					0.010 ± 0.001	0.020 ± 0.005		0.030 ± 0.001				0.024 ± 0.005*	0.010 ± 0.001*
Methyl (9E,12E)-octadeca-9,12-dienoate	2462-85-3									0.030 ± 0.014				
(9Z,12Z)-octadeca-9,12-dien-1-ol	506-43-4				0.010 ± 0.001						0.037 ± 0.005	0.040 ± 0.001		
[(2Z)-3,7-dimethylocta-2,6-dienyl] propanoate	105-91-9	Fruity	Medium	Green								0.500 ± 0.014		
Octadec-1-ene	112-88-9								0.010 ± 0.001					
[(Z)-dec-3-enyl] acetate	81634-99-3				0.037 ± 0.005		0.040 ± 0.005		0.010 ± 0.001		0.064 ± 0.005*	0.177 ± 0.005*		
[(Z)-dodec-5-enyl] acetate	16676-96-3					0.077 ± 0.005								
Cyclodecene	3618-12-0												0.024 ± 0.005	
Methyl ester 3,6-dodecadienoic acid	16106-1-7				0.010 ± 0.001*	0.117 ± 0.005*	0.050 ± 0.001*	0.160 ± 0.008*	0.010 ± 0.001*	0.250 ± 0.012*		0.147 ± 0.005	0.010 ± 0.001	0.010 ± 0.001
2-[(2R,5S)-5-ethenyl-5-methyloxolan-2-yl]propan-2-ol	5989-33-3	Earthy	Medium		0.007 ± 0.005		0.010 ± 0.001		0.010 ± 0.001			0.014 ± 0.005	0.017 ± 0.005*	0.003 ± 0.005*
Cyclotridecanone	832-10-0					0.033 ± 0.005				0.020 ± 0.001	0.020 ± 0.001			
[(2Z)-3,7-dimethylocta-2,6-dienyl] 2-methylpropanoate	2345-24-6	Fruity	Medium	Fruity				0.010 ± 0.001						
Methyl (Z)-octadec-6-enoate	2777-58-4							0.020 ± 0.005						
2-hexadecyloxirane	7390-81-0											0.034 ± 0.005		
(1S)-4,7-dimethyl-1-propan-2-yl-1,2-dihydronaphthalene	21391-99-1	Woody	Medium			0.010 ± 0.001					0.040 ± 0.001	0.014 ± 0.005		
3-[(3E)-4,8-dimethylnona-3,7-dienyl]furan	23262-34-2					0.010 ± 0.001					0.007 ± 0.005			
Alpha-dehydro-ar-himachalene	78204-62-3												0.027 ± 0.005	
7-methyl-4-methylidene-1-propan-2-yl-2,3-dihydro-1H-naphthalene	50277-34-4				0.087 ± 0.012		0.040 ± 0.005*	0.030 ± 0.001*	0.070 ± 0.005*	0.010 ± 0.005*			0.127 ± 0.012*	0.020 ± 0.001*
(2Z,6E)-3,7,11-trimethyldodeca-2,6,10-trien-1-ol	3790-71-4								0.010 ± 0.005		0.007 ± 0.005		0.030 ± 0.008	
(1E,5E)-1,5-dimethyl-8-propan-2-ylidenecyclodeca-1,5-diene	15423-57-1	Woody						0.020 ± 0.001						
(5S,6R,7S,10R)-7-Isopropyl-2,10-dimethylspiro[4.5]dec-1-en-6-ol	72203-99-7				0.010 ± 0.001									
(9Z,12Z)-octadeca-9,12-dien-1-ol	506-43-4				0.017 ± 0.005			0.020 ± 0.008						
Tridec-1-ene	2437-56-1							0.001 ± 0.001						
(8Z,11Z,14Z)-heptadeca-1,8,11,14-tetraene	10482-53-8	Costus										0.003 ± 0.001		
Tetradecan-2-one	2345-27-9							0.001 ± 0.001					0.047 ± 0.005	
2-ethylidene-1,7,7-trimethylbicyclo[2.2.1]heptane	62413-60-9												0.020 ± 0.001	
Cyclohexadec-5-en-1-one	37609-25-9	Musk	Medium			0.007 ± 0.005								
Isopentyl 8-methylnon-6-enoate	1215128-16-7								0.010 ± 0.001					
(Z)-2-pentadecen-4-yne	74646-33-6					0.070 ± 0.008		0.010 ± 0.001		0.090 ± 0.005		0.067 ± 0.005		
Methyl (6Z,9Z,12Z,15Z,18Z)-henicosa-6,9,12,15,18-pentaenoate	65919-53-1						0.010 ± 0.005*	0.020 ± 0.001*		0.030 ± 0.005		0.027 ± 0.005		
Nonadecan-2-one	629-66-3											0.030 ± 0.001		
Methyl (Z)-hexadec-7-enoate	56875-67-3											0.040 ± 0.001		
(9Z,12Z,15Z)-octadeca-9,12,15-trien-1-ol	506-44-5						0.020 ± 0.001		0.020 ± 0.001*	0.030 ± 0.005*	0.037 ± 0.005*	0.094 ± 0.005*	0.010 ± 0.001	
2-n-Butyl-2-cyclopentenone	5561_5-7												0.010 ± 0.001	
4,8,11,11-tetramethylbicyclo[7.2.0]undec-3-en-5-ol	913176-41-7								0.030 ± 0.005					
(6E)-3,7,11-trimethyldodeca-1,6,10-trien-3-ol	40716-66-3	Floral	Low	Green				0.001 ± 0.001						
1-cyclododecyl-ethanone	28925-0-0							0.010 ± 0.001						0.010 ± 0.001
(9Z,12Z,15Z)-octadeca-9,12,15-trien-1-ol	506-44-5							0.020 ± 0.001						0.020 ± 0.001
(1S,4R,4aS,8aR)-4,7-dimethyl-1-propan-2-yl-2,3,4,5,6,8a-hexahydro-1H-naphthalen-4a-ol	19912-67-5				0.020 ± 0.001		0.010 ± 0.001	0.010 ± 0.001	0.020 ± 0.005		0.010 ± 0.001		0.030 ± 0.001	0.010 ± 0.001
(1R,4R,6R,10S)-4,12,12-trimethyl-9-methylidene-5-oxatricyclo[8.2.0.04,6]dodecane	1139-30-6	Woody	Medium	Woody	0.177 ± 0.020*	0.013 ± 0.005*	0.030 ± 0.001*	0.020 ± 0.005*	0.060 ± 0.008*	0.040 ± 0.008*	0.060 ± 0.008	0.047 ± 0.017	0.247 ± 0.038	

(4Z,7Z)-1,5,9,9-tetramethyl-12-oxabicyclo[9.1.0]dodeca-4,7-diene	19888-33-6	Herbal			0.133 ± 0.012		0.020 ± 0.001*	0.010 ± 0.005*	0.030 ± 0.005*	0.010 ± 0.001*	0.040 ± 0.008	0.014 ± 0.005	0.137 ± 0.017	
Muurola-4,10(14)-dien-1.beta.-ol	257293-90-6							0.010 ± 0.012						
Neointermedeol	5945-72-2					0.027 ± 0.012			0.001 ± 0.001*	0.010 ± 0.005*				
(1R,3Z,7Z,11R)-1,5,5,8-tetramethyl-12-oxabicyclo[9.1.0]dodeca-3,7-diene	19888-34-7				0.740 ± 0.073		0.130 ± 0.005		0.230 ± 0.031*	0.080 ± 0.035*	0.244 ± 0.045*	0.080 ± 0.028*	0.837 ± 0.111	
(E,E,E)-2,6,10,14-hexadecatetraen-1-ol, 3,7,11,15-tetramethyl-acetate	61691-98-3				0.097 ± 0.012								0.120 ± 0.016	
3,7-Cycloundecadien-1-ol, 1,5,5,8-tetramethyl-	118014-38-3				0.010 ± 0.001				0.010 ± 0.001					0.020 ± 0.001
(1R,4S,4aR,8aR)-1,6-dimethyl-4-propan-2-yl-3,4,4a,7,8,8a-hexahydro-2H-naphthalen-1-ol	481-34-5	Herbal	Medium					0.010 ± 0.001	0.010 ± 0.001				0.057 ± 0.005	
(1S,4S,4aR,8aR)-1,6-dimethyl-4-propan-2-yl-3,4,4a,7,8,8a-hexahydro-2H-naphthalen-1-ol	5937_11-1	Balsamic					0.020 ± 0.008	0.030 ± 0.001						
2-[(3R,5R,6R)-6,10-dimethylspiro[4.5]dec-9-en-3-yl]propan-2-ol	1460-73-7							0.010 ± 0.001						
(1R,4S,4aR,8aS)-1,6-dimethyl-4-propan-2-yl-3,4,4a,7,8,8a-hexahydro-2H-naphthalen-1-ol	19435-97-3	Herbal	Medium		0.010 ± 0.001								0.007 ± 0.001	0.003 ± 0.001
2-[(2R,4aR,8aR)-4a,8-dimethyl-2,3,4,5,6,8a-hexahydro-1H-naphthalen-2-yl]propan-2-ol	473-16-5				0.010 ± 0.001			0.030 ± 0.005		0.010 ± 0.008				
(6Z,9Z,12Z,15Z)-Methyl octadeca-6,9,12,15-tetraenoate	73097-0-4								0.020 ± 0.009		0.010 ± 0.001	0.030 ± 0.008		
Methyl (8Z,11Z,14Z,17Z)-icosa-8,11,14,17-tetraenoate	132712-70-0						0.010 ± 0.001*	0.060 ± 0.005*	0.010 ± 0.005*	0.030 ± 0.008*		0.040 ± 0.014		
1,6-dimethyl-4-propan-2-ylnaphthalene	483-78-3												0.016 ± 0.005*	0.067 ± 0.028*
Caryophylla-4(12),8(13)-dien-5.alpha.-ol	19431-79-9												0.010 ± 0.008*	0.001 ± 0.001*
Methyl (Z)-5,11,14,17-eicosatetraenoate	59149-1-8				0.023 ± 0.005		0.080 ± 0.009*	0.050 ± 0.014*	0.090 ± 0.012*	0.020 ± 0.022*	0.080 ± 0.014*	0.017 ± 0.005*	0.010 ± 0.005	
Methyl ester docosapentaenoic acid	108698-2-8				0.010 ± 0.001		0.001 ± 0.005	0.001 ± 0.005		0.001 ± 0.001	0.007 ± 0.005	0.003 ± 0.005	0.323 ± 0.070	
Humulenol-II	19888-0-7				0.250 ± 0.057					0.001 ± 0.005				
[(4E)-11,11-dimethyl-8-methylidene-4-bicyclo[7.2.0]undec-4-enyl]methanol	50277-33-3				0.023 ± 0.009									
Methyl 2-(3-oxo-2-pentylcyclopentyl)acetate	24851-98-7	Floral	Medium	Floral	0.020 ± 0.001									

*Statistical difference between values ($p \leq 0.05$)
